# The Beneficial Effects of Natural Extracts and Bioactive Compounds on the Gut-Liver Axis: A Promising Intervention for Alcoholic Liver Disease

**DOI:** 10.3390/antiox11061211

**Published:** 2022-06-20

**Authors:** Liang Zhao, Shaoxuan Wang, Nanhai Zhang, Jingxuan Zhou, Arshad Mehmood, Rifat Nowshin Raka, Feng Zhou, Lei Zhao

**Affiliations:** 1 Beijing Advanced Innovation Center for Food Nutrition and Human Health, Beijing Engineering and Technology Research Center of Food Additives, School of Food and Health, Beijing Technology and Business University, Beijing 100048, China; liangzhao@btbu.edu.cn (L.Z.); 2002020204@st.btbu.edu.cn (S.W.); 10011521605@st.btbu.edu.cn (A.M.); 1870201018@st.btbu.edu.cn (R.N.R.); 2 Beijing Key Laboratory of Functional Food from Plant Resources, College of Food Science and Nutritional Engineering, China Agricultural University, Beijing 100083, China; nanhaizhang@cau.edu.cn (N.Z.); zjx888@cau.edu.cn (J.Z.); 3 Department of Food Science and Technology, University of Haripur, Haripur 22620, Pakistan

**Keywords:** bioactive compounds, gut-liver axis, alcoholic liver disease, gut microbiota

## Abstract

Alcoholic liver disease (ALD) is a major cause of morbidity and mortality worldwide. It can cause fatty liver (steatosis), steatohepatitis, fibrosis, cirrhosis, and liver cancer. Alcohol consumption can also disturb the composition of gut microbiota, increasing the composition of harmful microbes and decreasing beneficial ones. Restoring eubiosis or preventing dysbiosis after alcohol consumption is an important strategy in treating ALD. Plant natural products and polyphenolic compounds exert beneficial effects on several metabolic disorders associated with ALD. Natural products and related phytochemicals act through multiple pathways, such as modulating gut microbiota, improving redox stress, and anti-inflammation. In the present review article, we gather information on natural extract and bioactive compounds on the gut-liver axis for the possible treatment of ALD. Supplementation with natural extracts and bioactive compounds promoted the intestinal tight junction, protected against the alcohol-induced gut leakiness and inflammation, and reduced endotoxemia in alcohol-exposed animals. Taken together, natural extracts and bioactive compounds have strong potential against ALD; however, further clinical studies are still needed.

## 1. ALD: Epidemiology, Progression, Pathogenesis, and Treatment

Alcohol abuse is the fifth leading cause of disease and death worldwide. Around 2.4 billion people drink alcohol globally, including 1.5 billion (1.4–1.6) men and 0.9 billion (0.8–1.0) women [1]. However, drinking condition differs from country to country. For instance, the total per capita intake of alcohol in France is 12–13 L/adult, followed by the United Kingdom, Eastern Europe, United States of America, Italy, and North Africa/Middle East of 11–12 L/adult, 11–13 L/adult, 10 L/adult, 7 L/adult, and only 0–2 L/adult, respectively [2]. 

Alcohol is a major hazard of alcoholic liver disease (ALD), which begins with fatty liver disease (steatosis) and later progresses to alcoholic steatohepatitis (ASH) and fibrosis [3,4]. Distinguishing between acute and chronic alcohol intake and its impact on ALD prognosis is critical [5]. Although alcohol intake is the main cause of ALD, environmental variables and genetics may contribute to its progression [6]. Steatosis is one of several initial manifestations of ALD, essentially defined by an oversized liver. ALD can lead to serious consequences, such as cirrhosis and hepatocellular carcinoma, and there are currently no FDA (Food and Drug Administration)-approved treatments [4]. 

Despite steatosis being prevalent in ALD, the development of cirrhosis is observed in only 10% to 15%, whereas the incidence of ASH was reported in up to 20–40% of individuals [7,8]. Each year, around 2 million individuals die from liver failure worldwide, and alcohol consumption is responsible for up to 50% of cirrhosis mortality. 

In 2010, the global rate of death from alcohol-related cirrhosis was 7.2 per 100,000 people, which includes 9.7 in 100,000 males and 4.6 in 100,000 females [9]. So far, our expertise regarding the underlying pathogenesis of ALD is quite limited [10]. Liquor is indeed a primary hepatotoxin, and its consumption initiates a cascade of metabolic reactions that contribute to the final hepatotoxic consequence [11]. 

The preliminary clarification of malnutrition as the primary pathogenic pathway has been superseded by the current concept that alcohol is metabolized by the hepatocyte, initiating pathogenic processes that require the yield of peptides and cytokine production, immunology action, and oxidative damage [12]. In certain situations, the duration of liver damage is proportional to the amount of alcohol consumed [13]. With prolonged alcohol intake, alcohol-induced liver diseases can develop into infections, such as steatohepatitis, fibrosis, cirrhosis, and possibly hepatocellular carcinoma (HCC). Alcoholic-induced fatty liver is an alcohol-related disease in which alcohol promotes fat storage in liver cells [6]. If patients do not receive any treatment, hepatic fibrosis/cirrhosis or liver failure may occur [14]. Excessive alcohol intake can also cause steatohepatitis, which causes varying degrees of liver damage, such as steatosis, blistering, alcohol foam degeneration, lobular/fibrous inflammation, and acute cholestasis [15]. Alcoholic hepatitis (AH) is an acute inflammatory liver disease with high morbidity and mortality. Notably, AH was not associated with alcohol dose. AH is directly related to liver dysfunction and hepatic duct formation [16]. HCC is the third leading cause of cancer-related death in the world. Alcohol consumption can lead to oral, bowel, and liver cancers. Alcohol plays an important role in causing cancer by increasing the expression of many oncogenes, leading to cancer-causing mutations [6]. Addressing alcohol-induced liver injury requires an understanding of the complicated interaction of numerous distinct hepatic cell types [17,18]. 

The liver-gut microbiota axis involved reciprocal processes, including genetic, nutritional, and environmental variables. Modulation of the intestinal barrier explains the relationship between the gut and liver, occasionally with adverse consequences for the liver. Alcohol directly acts on liver parenchymal cells during liver pathogenesis, causing changes in intestinal barrier function, alteration of the microbiota, and enhancement of toll-like receptors (TLRs) activation in hepatic cells. Particular attention should be paid to modifying the gut flora, which contributes to the pathogenesis of liver disorders [19]. 

Modern therapeutics for ALD involve abstinence from alcohol, use of corticosteroids, s-adenosylmethionine, pentoxifylline, specific anti-TNF-α therapy, and type of diet. However, these therapies have no significant effect on fighting ALD. Therefore, lifestyle intervention on diet and exercise becomes the primary recommendation for ALD subjects [3,20,21]. Liver transplantation is a last resort, although invasive and expensive, and remains the therapeutic option when all other techniques fail to ameliorate the disease, usually only when patients abstain from alcohol [22,23]. Earlier, several researchers reported that natural extracts and bioactive compounds might be an ideal option for the prevention and treatment of many diseases, including ALD [24,25,26,27,28,29,30,31,32]. The preliminary purpose of the current review is to provide in-depth information regarding the effects of natural extracts and bioactive compounds against ALD via the gut-liver axis. This review will help ALD investigators understand nutritional therapy in regard to ALD.

## 2. Effect of Alcohol on Gut Microbiota

**Figure 1:**
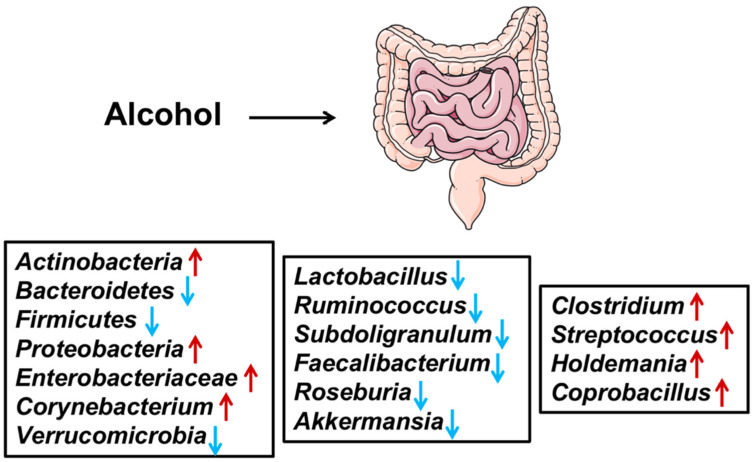
Alcohol abuse significantly affects many microbes in the gut. ↑, the abundance was upregulated; ↓, the abundance was down-regulated.

Alcohol abuse significantly affected many microbes in various parts of the gastrointestinal tract (Figure 1). The extent of small intestine bacterial overgrowth was reported to increase after alcohol exposure [33,34]. Moreover, many key phyla, such as *Actinobacteria*, *Bacteroidetes*, *Firmicutes*, and *Proteobacteria*, are affected by alcohol. The increment in the abundance of *Proteobacteria* phylum in response to alcohol was also documented [35,36]. The abundance of *Enterobacteriaceae* increased, while the abundance of *Bacteroidetes* decreased [37,38].

The relative abundance of *Corynebacterium* and *Actinobacteria* phylum was reported to increase after alcohol exposure, whereas phylum *Firmicutes* decreased [37,39]. The phylum *Firmicutes* contains many genera, namely *Lactobacillus*, *Ruminococcus*, *Subdoligranulum*, *Faecalibacterium*, and *Roseburia* that were decreased, whereas *Clostridium*, *Streptococcus*, *Holdemania*, and *Coprobacillus* increased after alcohol exposure [35,36,38,40,41]. The phylum *Verrucomicrobia*, which contains the *Akkermansia* genus, has decreased in the stool of people exposed to alcohol [36,42].

Collectively, chronic alcohol administration or intake results in dysbiosis, which is related to a decrease in beneficial bacteria. Restoring eubiosis or preventing dysbiosis after alcohol intake is an important strategy against ALD.

## 3. Mechanisms of Dysbiosis Driving Alcohol-Related Liver Diseases

### 3.1. Dysregulation of Bile Acid Metabolism

One of the significant communicators between the intestine and liver is bile acids. The hepatic biliary system secretes conjugated bile acids, which are converted as needed by intestinal bacteria in the duodenum [43]. Then the modified bile acids enter the enterohepatic circulation and reach the liver again. As previously mentioned, liver cirrhosis lowers normal bile flow in patients [44]. Bile acids stimulate the farnesoid X receptor in intestinal epithelial cells, leading to the induction of antimicrobial molecules [45]. On the contrary, the overgrowth of intestinal bacteria results from decreased bile flow. The experimental model of alcohol feeding in rodents showed a correlation between bile acid metabolism and the intestinal microbiome. Due to ethanol intake, taurine-conjugated bile acids were reduced in rats’ intestines and livers [46]. Nevertheless, the level of glycine-conjugated and unconjugated bile acids increased [47]. The partial reason behind this could be the overgrowth of gastrointestinal bacteria as cirrhosis patients exhibit escalated bile acids deconjugation [48]. Chronic abuse of alcohol in patients raises the total amount of bile acids, secondary bile acids, lithocholic acid, deoxycholic acid, and secondary-to-primary bile acid ratio in the stool [49]. If any patient develops advanced cirrhosis, they show an increment in serum level of conjugated bile acids and a reduction in the amount of total bile acids [49,50]. The secretion dimension of bile acid in the intestine of cirrhotic patients may be the underlying cause of both phenomena [44]. For a better understanding of pathogenesis related to chronic alcohol abuse and to develop potential therapeutic agents, extensive research is needed to further explore the interactions between bile acids and gut microbiota. This bidirectional crosstalk can better define the communications between the liver and intestine.

### 3.2. Microbial Products Contribute to Liver Inflammation and Disease

The liver readily absorbs toxins from the portal vein circulation as the unadulterated intestinal products reach the liver first. The promoters of hepatocellular injury are microbial toxins; including microbial pathogen-associated molecular patterns (PAMPs), fungal exotoxins (such as candidalysin), bacterial exotoxins (such as cytolysin secreted by *Enterococcus*), bacterial endotoxins (such as lipopolysaccharide [LPS] from gram-negative bacteria), hepatic toll-like receptors activated by endotoxins, and PAMPs that directly interact with pattern-recognition receptors present on hepatic stellate and Kupffer cells. The microbial products can advance cytokine stimulation, fibrotic changes, and oxidative stress (OS) of the inflammatory cascade [51].

Specific exotoxins exert pathogenicity in ALD patients. Compared to heavy drinking control, patients with AH show an increased abundance of *Enterococcus faecalis*, a cytolysin-producing bacteria. The quantity of cytolysin is associated with both the mortality and extremity of disease, the same as the fungal exotoxin candidalysin, which is also found in higher concentrations in AH patients [52]. Ethanol-comprising diet worsened liver injury in candidalysin-producing *Candida* colonized mice [53].

Studies have shown that dysbiosis may be related to the amount of endotoxins that circulate freely. Dysbiosis in ALD patients, along with AH and alcohol-related cirrhosis patients, revealed a correlated upsurge in flowing LPS [54,55,56]. Alcohol-related cirrhosis seemed to show a greater degree of endotoxemia than non-alcohol-induced cirrhosis, despite the end-stage liver disease scores being irrelevant [54]. Intestinal permeability is probably a vital implementer of endotoxemia. Markedly, half of the patients with ALD (about half of alcohol use disorders patients) showed increased permeability in the intestinal barrier, revealing a close association with microbiome changes. Therefore, increased intestinal permeability caused by microbial dysregulation is an important prerequisite for the progression of ALD [57,58].

### 3.3. Short-Chain Fatty Acids (SCFAs)

There are numerous processes involved in the regulation of intestinal permeability. Consumption of alcohol can affect many of those processes. Chronic alcohol intake deteriorates dysbiosis and subsequently disturbs the integrity of the tight junctions of the enterocyte, as SCFA-producing commensals are involved in maintaining barrier integrity [51]. Additionally, hepatic inflammation and adiposity can be mitigated by SCFAs [59]. ALD patients exhibited a consistent decrease in the microbiomes of *Lachnospiraceae* and *Ruminococcaceae* families [42,50,54,60,61,62]. Conversely, *Veillonella* is also known to produce SCFAs and is often expanded in patients with ALD [50,60,62,63]. There was a significant reduction in SCFAs in the feces of AH patients compared to heavy drinkers, despite changes in specific microbial patterns [60]. In short, intestinal permeability increases with the fading production of SCFAs, ultimately leading to hepatic inflammation.

### 3.4. Endotoxin

Endotoxin is one of the main components of the outer membrane of the gram-negative bacteria cell wall. Compared to non-alcoholic subjects, plasma of alcohol abused patients contained a 5-fold higher concentration of endotoxin [64]. Intake of alcohol disturbs the intestinal barrier functions and magnifies intestinal permeability. In the rat model, alcohol administration enabled systemic translocation and absorption of endotoxin [65]. The intensity of ethanol-induced liver injury in rats was significantly interrelated with endotoxin levels in plasma [66]. Endotoxin can cross the intestinal barrier and activate the Kupffer cells that generate TNF-α and superoxide in the liver, resulting in severe hepatic damage [67].

## 4. Protective Effect of Natural Products and Their Bioactive Compounds against Alcoholic Induced Gut Microbiota Dysbiosis

### 4.1. Bioactive Compounds against Alcohol-Induced Gut Microbiota Dysbiosis

Phenolic compounds are a large group of chemicals, such as phenolic acids, flavonoids, stilbenes, lignans, and other chemicals, commonly present in various edible plants. Phenolic compounds possess countless health benefits, including the hepatoprotective effect [26,27]. Earlier, Yuan et al. reported that epigallocatechin-3-gallate (EGCG) protected ALD via inhibiting alcohol-induced gut leakiness and inflammatory factors expressions, and reducing endotoxemia in rats [26]. Later, another study documented that EGCG acted as a prebiotic for *L. plantarum*, developing microbead synbox, and was promising as a therapeutic option for the ALD [28]. Similarly, other polyphenolic compounds, puerarin and kaempferol, alleviated ALD in mice via regulating intestinal tight junctions and inhibiting endotoxin leakage [29,30].

Earlier, tributyrin supplementation was found to protect mice from alcohol via expression and co-localization of tight junction (TJ) proteins (ZO-1, occludin), as well as butyrate receptor (GPR109A) and transporter (SLC5A8) in the ileum and proximal colon [68]. Aplysin, a brominated sesquiterpene compound purified from red alga Laurencia tristicha was studied against ALD. The daily treatment of aplysin (150 mg/kg bw) for 12 weeks markedly modulated the composition of *Escherichia coli*, *Bacteroides fragilis*, *Lactobacillus*, *Bifidobacterium*, and other key biomarkers, thus protecting ALD [69].

Astaxanthin was also reported to protect ALD via modulating mouse gut microbiota, such as decreasing the *Bacteroidetes*, *Proteobacteria*, *Parabacteroides*, *Butyricimonas*, *Bilophila* and increasing *Akkermansia* and *Verrucomicrobia* in mice [31].

Berberine is a natural compound present in many plant extracts and possesses multiple health effects. Recently, Li et al. conducted a study to explore the protective effect of berberine against alcohol-mediated gut microbiota dysbiosis. Berberine (10, 50, and 100 mg/kg bw) was orally administrated to the mice for 33 days. Results revealed that berberine treatments markedly improve gut microbiota dysbiosis by increasing the abundance of *Akkermansia muciniphila* [32]. More recently, Han et al. investigated the protective effect of cornel iridoid glycoside isolated from *Cornus officinalis* Sieb. et Zucc against ALD. The cornel iridoid glycoside was supplemented at the dosage of 50, 100, and 200 mg/kg bw for 16 days in the mice. The results revealed that cornel iridoid glycoside supplementation significantly attenuated ALD via enhancing antioxidant activities, reducing inflammation, and altering intestinal microbial diversity. Cornel iridoid glycoside supplementation increased the abundance of *Lactobacillus* and decreased the proportion of *norank_f_Muribaculaceae* and *norank_f_Desulfovibrionaceae* in mice [70]. Furthermore, ursolic acid and antrodin A have recently been reported in two different studies to protect against alcohol-induced liver injury via the gut-liver axis [71,72].

Polysaccharides are polymeric carbohydrate molecules abundantly present in various plants, algae, microorganisms, and animals, exerting a wide array of biological activities, including hepatoprotective activities [73]. Many authors reported that polysaccharides could protect the liver from alcohol damage via multiple pathways, including restoring gut dysbiosis [32,74,75,76]. In detail, Wang et al. isolated polysaccharides from garlic (molecular weight: 10 Kda, acid heteropolysaccharide), which was further studied against ALD in mice. Results showed that daily garlic polysaccharide administration (150 and 250 mg/kg bw for 30 days) could alleviate various biochemical indicators, increasing the abundance of *Lachnospiraceae* and *Lactobacillus*, and decreasing the abundance of *Facklamia* and *Firmicutes* in ethanol-induced mice [74]. Yang et al. found that inulin administration could ameliorate ALD via inhibiting the LPS-TLR4-Mψ axis, and rectified gut dysbiosis mainly by increasing the abundance of *Lactobacillus*, *Lactococcus*, and *Allobaculum* and reducing the abundance of *Parasutterella* [75]. In another study, *Coprinus comatus* polysaccharides could regulate gut microbiota in ALD mice by increasing the proportion of *Lachnospiraceae*, *Firmicutes*, *Muribaculaceae*, and *Bacteroidetes* and decreasing the *Rikenellaceae* proportion, which showed prebiotic-like effects on the intestinal flora in ALD mice [32]. Similarly, *Wolfiporia cocos* polysaccharides were also reported to modulate gut microbiota in ALD mice, mainly by increasing the *Firmicutes* to *Proteobacteria* ratio and the abundance of *Lachnospiraceae* [77]. Oral administration of oyster (*Crassostrea gigas*) polysaccharides (282 mg/kg bw) could also increase the proportion of *Roseburia* spp. and *Lactobacillus reuteri*, and decrease *Escherichia* proportion in ALD mice [78].

### 4.2. Natural Product Extracts against Alcohol-Induced Gut Microbiota Dysbiosis

#### 4.2.1. Fruits and Vegetables

Lychee (*Litchi chinensis* Sonn.) pulp extract rich in polyphenolic compounds (procyanidin B2, (-)-epicatechin, quercetin-3-O-rutinoside-7-O-α-L-rhamnosidase, rutin, and isorhamnetin-3-O-rutinoside) was orally given (0.2 and 0.4 g/L bw) to the ethanol-exposed (4%, v/v) mice for 8 weeks. Results revealed that compared with the ethanol group, lychee pulp extract supplementation increased the relative abundance of the *Lactobacillus* genus, *Bacteroides acidifaciens* species, *Actinobacteria* phylum, and *Coriobacteriaceae* family, whereas it decreased the abundance of *Dehalobacteriaceae* family and *Odoribacter* genus. Furthermore, it was also observed that lychee pulp extract supplementation could upregulate the expression of intestinal tight junction proteins, antimicrobial proteins, and mucus proteins while declining the serum endotoxin level. They concluded that lychee pulp extract has strong potential against alcoholic abuse [79]. In another study, pomegranate extract could also prevent intestinal apoptosis, endotoxemia, alcohol-induced intestinal leakage, and inflammation by regulating TJ/ adherent junction proteins [80]. *Corchorus olitorius* L., also known as molokhia, is a pantropical plant consumed as a vegetable in Africa and Eastern Asia that exerts a protective effect against several diseases [76,81]. Recently, Do et al. documented that administration of *molokhia* extract (50 and 100 mg/kg bw) restored the composition of *Muribaculum* and enhanced the intestinal barrier function in mice [76].

#### 4.2.2. Cereals

Tang et al. documented that oats supplementation (10 g/kg bw) for 12 weeks to the rats could prevent alcohol-induced intestinal leakage by protecting the integrity of tight junctions and colonic mucosa [82]. Similarly, supplementation with rice bran phenolic extract was also reported to combat alcohol-induced liver injury via alleviating intestinal microbiota dysbiosis. Briefly, rice bran phenolic extract supplementation increased the abundance of *Bacteroides acidifaciens* and *Lactobacillus*, whereas it decreased pathogenic bacteria such as *Muribaculum*. Furthermore, it was also observed that rice bran phenolic extract could protect the intestinal barrier from alcohol [83]. Recently, Yang et al. reported that wheat embryo globulin could maintain the composition of gut microbiota [84].

#### 4.2.3. Oils

Fish oil contains a significant amount of n-3 polyunsaturated fatty acids (PUFAs), which have been reported to alter gut microbiota. It has also been documented that fish oil supplementation can increase *Bifidobacterium* and decrease *Escherichia coli* in the feces of rats fed alcohol [85,86,87]. More recently, Chen et al. also reported that fish oil supplementation reduced the overgrowth of *Rikenellaceae*, *Bacteroidetes*, *Alistipes*, and *Bacillaceae*, inhibited endotoxin production, and suppressed TLR4 activation in chronic ethanol-fed rats [30].

Flaxseed oil was also reported to protect against the adverse effect of alcohol by modulating gut microbiota in alcohol-induced liver injury mice [88]. According to the report, *Decaisnea insignis* seed oil (containing palmitoleic acid, palmitic acid, and oleic acid) could protect against alcohol-associated liver damage in mice via increasing the abundance of *Lactobacillus*, *Ruminoccoceae_UCG_004* and decreasing *Parabacteroides* abundance [89].

Okra seed oil supplementation has been reported to improve ALD via regulating intestinal microbiota. Briefly, Okra seed oil supplementation at the dosage of 400 and 800 mg/kg bw for 8 weeks decreased the proportion of *Proteobacteria*, *Clostridium XlVa*, and *Staphylococcus*, while enhancing the abundance of *Bacteroidetes* in alcohol-treated mice [90].

#### 4.2.4. Tea

It has been reported that Pu-erh tea extract (PTE) played a protective role against ALD mainly through improving OS, lipid accumulation, inflammation, and microbiota dysbiosis. PTE treatment increased the relative abundance of potentially beneficial bacteria (*Bifidobacterium* and *Allobaculum*) and decreased the relative abundance of harmful bacteria (*Helicobacter* and *Bacteroides*) [91]. More recently, Li et al. studied the effects of six tea samples: two black teas (Dianhong tea and Yingde Black tea), two oolong teas (Tieguanyin Tea and Fenghuang Danzong Tea), and two dark teas (Fuzhuan Brick tea and Selenium-Enriched Dark tea) against ALD in mice. Results revealed that all tea sample supplementation markedly protected from the adverse effect of alcohol. However, more profound results were observed in oolong tea and dark tea. Moreover, their findings suggested that *Akkermansia* is the target microorganism for Tieguanyin Tea and Fu Brick Tea [92].

#### 4.2.5. Fermented Liquids

Vinegar is fermented acidic food rich in various bioactive compounds such as polyphenols, flavonoids, and melanoidins. It was previously reported that Shanxi aged vinegar extract (SAVE) contains chlorogenic acid, p-hydroxybenzoic acid, ferulic acid, rutin, syringic acid, gallic acid, and other polyphenols, which exerts high antioxidant activity and protects liver cells from oxidative damage [83]. In another study, the same research group documented that polyphenol-rich SAVE attenuated ALD via regulating gut microbiota. Briefly, they found that various microbes (*Lactobacillus*, *Bacteroidetes*, *Akkermansia*, *Verrucomicrobia*) showed a significant positive correlation with OS and inflammatory indictors (occludin, Reg3b, Reg3g, and ZO-1), whereas *Proteobacteria*, *Parabacteroides*, *Firmicutes*, *Bilophila*, and *Butyricimonas* exhibited the opposite effect [93]. In addition, this research group also investigated the protective effect of another vinegar (Zhenjiang aromatic vinegar; ZAV) against ethanol-induced liver injury. Results showed that ZAV could regulate the composition of gut microbiota and immune factors in ALD mice. Additionally, *Lachnospiraceae_NK4A136_group*, *Bacteroidetes*, and *Akkermansia* were positively correlated with antimicrobial peptides and intestinal immune factors, but negatively correlated with inflammatory and OS parameters [94].

Fermented rice liquors (called *Makgeolli* in Korea) were also documented to restore fecal microbiota compositions in mice induced by alcohol. The abundance of *Bacteroidetes* and *Firmicutes* phyla was observed to return to the control group level. Moreover, treatment with fermented rice liquors also increased the content of fecal SCFA and reduced inflammatory responses in mice induced by alcohol [95]. Baijiu, a Chinese traditional fermented liquor containing volatile compounds such as esters, acids, and phenols, was also reported to increase the relative abundance (11%) of *Lactobacillus* compared to the ethanol-treated group (1.80%) [96].

Ran et al. studied the protective effect of sea buckthorn-fermented liquid against ALD. The sea buckthorn was fermented with a *Lactobacillus plantarum* BNCC194165 strain, exhibiting a significant increment in the total flavonoids, total triterpenes, and SCFAs compared with the unfermented sea buckthorn. Furthermore, fermented sea buckthorn liquid was sterilized and orally given (1.75, 2.675, and 5.35 g/kg bw) to the mice for 15 days. Results showed that fermented sea buckthorn liquid could protect the liver from alcohol by improving OS, decreasing inflammation, and regulating gut microbiota. The high dosage of fermented sea buckthorn liquid (5.35 g/kg bw) significantly enhanced the abundance of *Lactobacillus* and decreased the abundance of *Ruminiclostridium*, *Akkermansia*, *Alistipes*, and *Turicibacter* in mice [97].

#### 4.2.6. Herbs and Miscellaneous Extracts

Rhubarb (*Rheum palmatum* and *Rheum officinale*), a natural edible herb, contains a variety of bioactive compounds, including anthraquinone derivatives with hepatoprotective effects [98]. Neyrinck et al. reported that rhubarb extract (0.3%) could change the microbial composition of *Akkermansia muciniphila* and *Parabacteroides goldsteinii*, improving hepatic injury and decreasing inflammatory and OS biomarkers in alcohol-induced mice [99].

The mixture of *Ginkgo biloba* and *Rosa roxburghii* juice, rich in bioactive compounds (rutin, quercetin, kaempferol, isorhamnetin, ginkgolide C, bilobalide, ginkgolide A, and ginkgolide B), was reported to protect alcoholic intestinal barrier dysfunction via restoring tight junctions [100]. In another study, *Lactobacillus fermentum* KP-3-fermented ginseng (*Panax ginseng*) was orally administrated (390 mg/kg bw) to alcohol-exposed mice for 14 days. Results revealed that fermented ginseng supplementation could improve gut microbiota dysbiosis via restoring the abundance of *Lactobacillus* and *Bifidobacteria*, *Bacteroidetes* phylum, and the *Proteobacteria* genus of the *Sutterella* phylum, *Verrucomicrobia* phylum, *Allobaculum* genus, *Ruminococcus* genus, *Adlercreutzia* genus, and *Actinobacteria* phylum [101].

Choi et al. conducted a study to explore the protective effect of defatted Tenebrio molitor larva fermented extract against chronic alcohol-fed rats. Results showed that defatted *Tenebrio molitor* larva fermented with *Saccharomyces cerevisiae* strain (KCTC 17299) extract at the dosage of 200 mg/kg/day could attenuate ALD via modulating intestinal microflora, steatosis, and inflammation. It was observed that defatted *Tenebrio molitor* larva extract restored the *Lactobacillus johnsonii* abundance [102]. Similarly, many other natural extracts such as *Pogostemon cablin*, edible insect *Gryllus bimaculatus*, Semen Hoveniae, and *Dendropanax morbifera* leaf extract were reported to protect against alcohol-mediated gut microbiota dysbiosis [103,104,105,106].

## 5. Discussion

**Figure 2:**
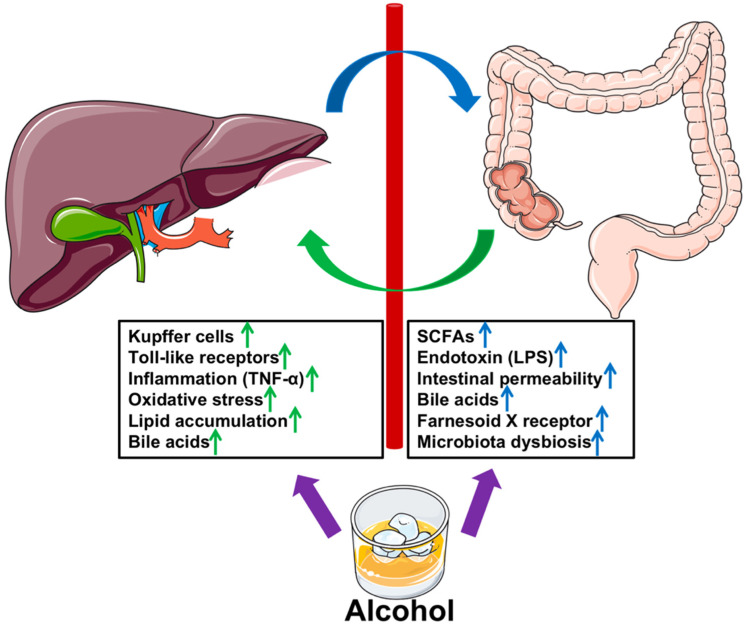
Summary of the effects of chronic alcohol consumption on the gut-liver axis.

A large amount of evidence suggests that intestinal microbiome dysregulation is a key risk factor for the progression/development of ALD. The graphical summary of the effects of chronic alcohol consumption on the gut-liver axis is shown in Figure 2. Prevention of alcohol-induced dysbiosis is an important strategy in the treatment of ALD. In this regard, natural products and bioactive compounds play a significant role. As mentioned above, edible plants and their bioactive compounds could restore gut microbiota in animal models (Table 1).

Briefly, *Lactobacillus*, a therapeutically relevant bacterial genus, decreased after alcohol intake, which can be further restored through various bioactive compounds and natural products [70,74,78,79,96,97]. *Lactobacillus* is a beneficial bacteria that produces bacteriocins such as antibiotics, which further inhibit harmful microbes of the *Enterobacteriaceae* family, such as Salmonella or Shigella [107]. *Lactobacillus* can protect against pathogenic and invasive bacteria by adhering to intestinal epithelial cells [108,109]. In addition, they produce SCFAs (lactic acid, propionic acid, or butyric acid), which provide nutrition to epithelial cells [110].

*Allobaculum* and *Bifidobacterium* are beneficial intestinal bacteria that produce SCFAs (butyric and lactic acids) and a small amount of ethanol from glucose. In addition, *Bifidobacterium* is a potential acetaldehyde accumulator [101]. Natural products and bioactive compounds increased the abundance of *Allobaculum* and *Bifidobacterium* in alcohol-exposed mice [91].

The *Bacteroidetes* phylum is composed of three major classes of gram-negative bacteria present in the intestines, upper respiratory tract, mouth, and genital tracts of animals and humans, exerting both beneficial and harmful functions. *Bacteroidetes* may lead to endogenous infections due to microecological imbalance. It has been documented that *Bacteroides fragilis* can produce polysaccharide A to relieve colitis in animals [111]. Conversely, it also produces toxins, which facilitate pro-carcinogenic effects and mediate colon tumorigenesis [112]. Moreover, the growth of *Proteobacteri* (pro-inflammatory intestinal microbes) increased due to imbalanced microbial composition, linked with the occurrence and development of disease [113]. Natural products and bioactive compounds were reported to decrease the abundance of *Bacteroidetes* and *Proteobacteria* in alcohol-exposed animals [31].

Several studies have documented the beneficial effects of *Akkermansia* on host metabolites. In the phylum *Verrucomicrobia*, *Akkermansia* is a dominant genus that interferes with intestinal mucin, enhances gut barrier function, increases mucus thickness, and inversely correlates with metabolic syndrome and inflammation [114,115,116]. *Akkermansia* deficiency is an early sign of alcoholic gut dysbiosis [117]. Furthermore, alcohol exposure reduced the population of *Akkermansia* in both mice and humans, and alcoholic fatty liver disease (AFLD) can be improved by supplementation of the genus, indicating the protective role of this bacterium against AFLD [118]. Due to the nature of probiotics, the abundance of *Akkermansia* may be affected by dietary ingredient supplementation [119]. On the other hand, the abundance of *Staphylococcus* was directly related to the expression of TNF-α in the liver of ALD, and the overabundance of *Staphylococcus* in the gut may be linked with the aggravation of hepatic inflammation [120]. Bioactive compounds could decrease the *Helicobacter* abundance, a key marker in patients with gastric disease multiplying and growing in the intestine, slowing its production of a huge amount of endotoxin in the gut [121,122].

Although many studies support that natural products and bioactive compounds could modulate gut microbiota in animal experiments, there are still some limitations in current studies, such as that (a) some plant-based functional food extracts are reported to exert a protective effect against ALD dysbiosis. However, their bioactive components have not yet been characterized. (b) the dosage needs to be optimized to avoid adverse effects and contribute to the beneficial effects of plant-based functional foods, (c) effects of plant-based functional foods and their bioactive components are mostly investigated based on animal models, which lacks the in-depth systematic analyses, (d) large-scale clinical trials investigating the role of plant-based functional foods and their bioactive components against ALD have not been conducted as results based on animal models and humans may differ. Despite limitations and gaps, plant-based functional foods and their bioactive components are a viable approach for treating ALD dysbiosis.

## 6. Conclusions

ALD is a disease caused by excessive consumption of alcohol with high morbidity and mortality worldwide. Gut microbiota plays a key role in many metabolic processes beneficial to the host, such as the production of SCFAs and vitamins. However, excessive intake of alcohol is also associated with gut dysbiosis in the pathogenesis of ALD. Natural products and phytochemicals are important sources of novel therapeutic agents against chronic diseases, including ALD. Natural products and related phytochemicals act through multiple pathways, such as modulating gut microbiota, improving redox stress, and anti-inflammation. Natural products and phytochemicals can increase the relative abundance of beneficial microbes (*Lactobacillus*, *Bacteroides acidifaciens*, *Actinobacteria*, *Coriobacteriaceae*, *Akkermansia*, *Verrucomicrobia*, etc.) and decrease the relative abundance of harmful microbes (*Bacteroidetes, Proteobacteria, Parabacteroides, Butyricimonas, Bilophila,* etc.), indicating the protective effects against ALD. Natural products could also prevent intestinal apoptosis, endotoxemia, alcohol-induced intestinal leakage, and inflammation by regulating TJ/adherent junction proteins, LPS-TLR4 pathway, and OS biomarkers, thereby protecting against ALD. Based on animal studies, natural products and related phytochemicals have proved ideal candidates for combating ALD and its complications. Notably, the transferability of these findings might not yet be possible because clinical trials are still warranted.

## Figures and Tables

**Table 1:** Summary of the protective effect of various natural products against ALD.

Extract	Bioactive Compound	Study Design	Major Finding	Ref
*Litchi chinensis* Sonn	Procyanidin B2, (-)-epicatechin, quercetin-3-O-rutinoside-7-O-α-L-rhamnosidase, rutin, and isorhamnetin-3-O-rutinoside	Lychee pulp extract was orally given (0.2 and 0.4 g/L bw) to the ethanol-exposed (4%, *v*/*v*) mice for eight weeks.	Lychee pulp extract supplementation upregulated the expression of intestinal tight junction proteins, antimicrobial proteins, and mucus protecting proteins while decreasing the serum endotoxin level.	[79]
Pomegranate	Not investigated	Age-matched 7-week-old female Fischer 344 wild-type rats were orally administered a daily dose of 600 mg pomegranate extracts/kg, based on the safety and effective dosages of pomegranate extract binge alcohol (5 g/kg/dose).	Pomegranate extract could protect ALD via modulating TJ/AJ proteins, preventing elevated apoptosis of enterocytes, endotoxemia, alcohol-induced gut leakiness, and inflammation.	[80]
*Corchorus olitorius* L.	Chlorogenic acid, catechin, and astragalin	Mice were orally administered 40% ethanol (4.0 g/kg/day) and 50 or 100 mg/kg of *Corchorus olitorius* L. extract, respectively.	*Corchorus olitorius* L. extract (50 and 100 mg/kg bw) administration restored *Muribaculum* composition and protected gut barrier function in mice.	[76]
Oats	Not investigated	Male SD rats were gavaged for 12 weeks with alcohol (starting dose of 1 g/kg increasing to 6 g/kg/day over the first 2 weeks) or dextrose, with or without oats supplementation (10 g/kg/day).	Oats supplementation (10 g/kg bw) could protect alcohol-induced leaky gut by protecting the integrity of tight junctions and colonic mucosa.	[82]
rice bran phenolic extract	Acacetin, protocatechuic aldehyde, caffeic acid, p-coumaric acid, ferulic acid, sinapic acid, quercitrin, vitexin, rutin, hesperidin, ethyl caffeate, and ethyl coumarate	Rats were given a control liquid diet, an ethanol (4%, *w*/*v*) liquid diet, and an ethanol (4%, *w*/*v*) liquid diet supplemented with 0.25 or 0.50 g/L rice bran phenolic extract for eight weeks, respectively.	Rice bran phenolic extract supplementation increased the Bacteroides acidifaciens and Lactobacillus population while decreasing pathogenic bacteria such as Muribaculum. Rice bran phenolic extract could protect the intestinal barrier function from alcohol.	[83]
Fish oil	Not investigated	Thirty-six male Wistar rats (8 weeks old) were divided into six groups: control, control diet with 25% fish oil substitution, control diet with 57% fish oil substitution, ethanol-containing diet, an ethanol-containing diet with 25% fish oil substitution, and ethanol-containing diet with 57% fish oil substitution groups.	Fish oil supplementation decreased overgrowth of *Rikenellaceae*, *Bacteroidetes*, *Alistipes*, and *Bacillaceae*, inhibited endotoxin production, and suppressed TLR4 activation in chronic ethanol-fed rats.	[30]
*Decaisnea insignis* seed oil	Palmitoleic acid, palmitic acid, and oleic acid	Fifty mice were orally administered with 38% alcohol (0.4 mL/day) and without or with Decaisnea insignis seed oil (3, 6, and 12 g/kg) for consecutive 12 weeks.	Decaisnea insignis seed oil increased the abundance of *Lactobacillus*, *Ruminoccoceae_UCG_004,* and decreased *Parabacteroides* abundance.	[89]
Okra seed oil	Linoleic acid, palmitic acid, oleic acid, decanoic acid, lauric acid, tridecanoic acid, myristic acid, palmitoleic acid, trans-9-octadecenoic acid, stearic acid, gamma-linolenic acid, eicosenoic acid, and behenic acid	Okra seed oil was orally given at the dosage of 400 and 800 mg/kg bw for 8 weeks to the alcohol-administered mice.	Okra seed oil supplementation decreased the proportion of *Proteobacteria*, *Clostridium XlVa,* and *Staphylococcus*, while enhancing the *Bacteroidetes* population in alcohol-treated mice.	[90]
Pu-erh tea extract	(−)-allocatechin, (−)-gallocatechin gallate, (−)-epicatechin, (−)-epicatechin gallate, (−)-epigallocatechin, (−)-epigallocatechin gallate, (−)-catechin, (−)-catechin gallate, γ-aminobutyric acid	Mice were orally given Pu-erh tea extract at the dosage of 0.1 or 0.4% (1 or 4 g/L, *w*/*v*) for 4 weeks.	PTE treatment increased the relative abundance of potentially beneficial bacteria (*Bifidobacterium* and *Allobaculum*) and decreased the relative abundance of harmful bacteria (*Helicobacter* and *Bacteroides*).	[91]
Green tea	Gallic acid, gallocatechin, epigallocatechin, catechin, chlorogenic acid, caffeine, epigallocatechin gallate, epicatechin, ellagic acid, myricetin, quercitrin, astragalin, theaflavin, and kaempferol	Green tea samples were given to the mice at a dosage of 200 mg/kg bw for 4 weeks.	*Akkermansia* is the target microbe for the protective effects of Tieguanyin Tea and Fu Brick Tea toward ALD.	[92]
*Tenebrio molitor* larva	Not investigated	The alcohol-fed rats were administered defatted *Tenebrio molitor* larva (50, 100, or 200 mg/kg/day) orally for eight weeks	Defatted Tenebrio molitor larva fermented with *Saccharomyces cerevisiae* strain (KCTC 17299) extract at the dosage of 200 mg/kg/day attenuated ALD via modulating intestinal microflora (restoring the *Lactobacillus johnsonii* abundance), steatosis, and inflammation.	[102]
Garlic polysaccharide	Acid heteropolysaccharide	The purified garlic polysaccharide was orally administrated at a dosage of 150 and 250 mg/kg bw for 30 days.	Daily garlic polysaccharide administration (150 and 250 mg/kg bw for 30 days) could alleviate various biochemical indicators, increasing the abundance of *Lachnospiraceae* and *Lactobacillus*, and decreasing the abundance of *Facklamia* and *Firmicutes* in ethanol-induced mice.	[74]
*Coprinus comatus* polysaccharides	Not investigated	*Coprinus comatus* polysaccharides (200 mg per kg bw) were orally administered for 30 days.	Coprinus comatus polysaccharides could regulate gut microbiota in ALD mice by increasing the proportion of *Lachnospiraceae*, *Firmicutes*, *Muribaculaceae*, and *Bacteroidetes,* and by decreasing the *Rikenellaceae* proportion, which showed prebiotic-like effects on the intestinal flora in ALD mice.	[32]
Oyster (*Crassostrea gigas)*	Not investigated	Oyster polysaccharides (282 mg/kg bw) were orally given to mice.	Oral administration of oyster (Crassostrea *gigas)* polysaccharides (282 mg/kg bw) could also increase the proportion of *Roseburia spp*. and *Lactobacillus reuteri*, and decrease *Escherichia* proportion in ALD mice.	[78]
